# Development and Evaluation of Culturally and Linguistically Tailored Mobile App to Promote Breast Cancer Screening

**DOI:** 10.3390/jcm7080181

**Published:** 2018-07-24

**Authors:** Hee Yun Lee, Mi Hwa Lee, Zan Gao, Karim Sadak

**Affiliations:** 1 School of Social Work, University of Alabama, Tuscaloosa, AL 35487, USA; 2 College of Health and Human Performance, East Carolina University, Greenville, NC 27858, USA; leemih17@ecu.edu; 3 School of Kinesiology, University of Minnesota, Minneapolis, MN 55455, USA; gaoz@umn.edu; 4 Masonic Cancer Center, University of Minnesota Medical School, Minneapolis, MN 55455, USA; ktsadak@umn.edu

**Keywords:** breast cancer, mammogram, mobile phone-based health intervention, mHealth, app, health navigator, Korean American immigrant women

## Abstract

Background: While a significant breast cancer burden exists for Korean American immigrant women, their cancer screening behavior is strikingly poor, and few interventions have focused on this population. To promote breast cancer screening behavior in Korean American immigrant women, a mobile phone multimedia messaging intervention (mMammogram) was developed. Objective: The current study explores the impact of mMammogram on changes to study participants’ screening behavior and proposes suggestions for how the intervention can be improved for wide dissemination and implementation in the Korean American community. Material and Methods: Data were collected through qualitative research methods. Three focus groups were conducted with 14 Korean immigrant women who completed the mMammogram. Findings: Three themes emerged: (1) better understanding of breast cancer and screening through mMammogram (e.g., increased knowledge on breast cancer and screening methods, increased understanding of the importance of regular mammography, and reduced anxiety about mammography); (2) health navigators as a trigger to promote mammography (e.g., providing resources for free or low-cost mammograms and scheduling mammogram appointments); and (3) suggestions for mMammogram (e.g., technical issues and program period). Conclusions: Mobile app intervention that is culturally tailored, along with health navigation services, can be a feasible, effective, and acceptable tool to promote breast cancer screening behaviors in underserved immigrant women. A mobile app can cover a broad range of breast cancer health topics and the health navigator can further help women overcome barriers to screening. A health navigation service is critical in overcoming language, transportation, and health accessibility barriers and triggering a positive change in their health screening behavior, especially for newly arrived immigrant populations.

## 1. Introduction

Although the cancer mortality rate is decreasing in the United States (U.S.) among the general population, the rate remains on the rise for Asian Americans, specifically for the Korean American population [1]. Studies report that Korean Americans have one of the highest overall cancer mortality rates across Asian American subgroups [2]. For example, breast cancer has been cited as both the most frequently diagnosed cancer and the most common cause of cancer deaths for Korean American women [3]. Cancer screening has been shown to be an effective measure in reducing cancer morbidity, contributing to the decline in cancer mortality rates in the general U.S. population [2,4]. However, there is a lack of utilization of these screening tools among Korean Americans. Studies of Asian American subgroups found that Korean Americans have had the lowest overall cancer screening rate [5], and Korean American women reported the lowest breast cancer screening rates [4,6,7,8].

Korean American women experience a variety of barriers to breast cancer screening. Studies on the topic have identified several obstacles to screening, including low socioeconomic status, language barriers, difficulties accessing healthcare (e.g., inadequate health insurance and burdens of cost and time), lack of cultural awareness by healthcare providers, lack of knowledge about screening guidelines, culture-based health beliefs (e.g., the belief that screening is unnecessary in the absence of symptoms), cultural modesty or embarrassment in terms of physical examinations, a fatalistic view of cancer, and fear of screening results [8,9,10,11,12]. Alongside barriers, the literature has also cited a number of facilitators that have been found to promote increased mammogram use in Korean American women. The noted factors include perceived benefits of mammograms, perceived self-efficacy, and perceived susceptibility to breast cancer [13,14].

A handful of interventions specifically designed to address barriers and promote breast cancer screening in the Korean American community have been implemented. These community-based efforts include peer-led workshops, education sessions, a lay health worker intervention, and distribution of print material about screening guidelines [13,15,16,17,18,19]. These interventions have only been partially effective in promoting screening among this population. Key reasons cited in the research literature for such limited success include the fact that Korean American women are a hard-to-reach population due to geographical dispersion across the U.S. [17] and that past efforts have utilized a “one size fits all” approach rather than tailored interventions that target specific obstacles individuals face [18,19]. A more efficacious approach may be to develop a culturally appropriate, personalized intervention that promotes breast cancer screening among Korean American women and responds to the systemic sociocultural barriers present in this population.

An innovative and promising solution might be a mobile phone-based health intervention. It is likely to provide low-cost and effective methods of contacting hard-to-reach populations with tailored individual messages, covering broad content areas while also overcoming restrictions to place and time of delivery [20]. Mobile health, or mHealth—described as “the delivery of healthcare services and information via mobile communication devices” [21]—is emerging as a direct and effective medium to change health behavior. mHealth is an element or expansion of eHealth [22,23]: it is the broader trend that incorporates any health service information delivered through the Internet or related technology [24]. The growing mHealth field has already proven to be effective in the realm of health behavior change [25,26,27].

To promote breast cancer screening behavior of Korean American immigrant women, the intervention program called mobile Mammogram (mMammogram) was developed. The current study aims to explore the perspectives of Korean American immigrant women regarding their use of mMammogram, and how the program (1) promotes knowledge and positive attitude toward breast cancer screening; (2) motivates them to get breast cancer screening; and (3) may be improved for wide dissemination and implementation in the Korean American community. The current study provides critical information for developing a culturally relevant and personally tailored intervention to promote mammograms among underserved immigrant women and other disadvantaged minority groups which can effectively improve breast cancer health equity. 

## 2. Materials and Methods

### 2.1. mMammogram

mMammogram was a 7-day mobile phone-based multimedia messaging program. mMammogram provided knowledge on breast cancer and screening methods along with guidelines, cultural barriers (e.g., fatalistic view and lack of preventive care concept), and information to access the healthcare system. This content was delivered in various formats of tailored messages using culture-specific emoticons, graphs, images, pictures, and videos in the Korean language. mMammogram also offered health navigation services, such as providing necessary resources (e.g., free or low-cost mammogram) and making an appointment for a mammogram. More detailed information about the mMammogram Randomized Clinical Trial (RCT) has been previously published [28].

### 2.2. Research Method and Data Collection

The research team utilized qualitative research methods to address the research aims. Three focus groups were conducted between June 2014 and February 2015 with a total of 14 Korean immigrant women who completed mMammogram prior to the focus groups. Six women participated in the first focus group, five in the second group, and three in the third group. Details of data collection (e.g., study participants’ inclusion and exclusion criteria and detailed recruiting process on mMammogram) are described elsewhere [28]. University of Minnesota’s Institutional Review Board approved this study.

The first author, along with two research staff members, facilitated and took notes during each focus group. The focus group interviews were semi-structured and included open-ended questions that asked participants about general feedback on (1) content, duration, and concerns of mMammogram; (2) changes in their knowledge, attitude, and motivation for screening receipt after participating in mMammogram; and (3) their screening experience if they had a mammogram after the intervention and their recommendation for dissemination in the Korean American community. Some examples of questions used in the focus group include: What are your perspective about the mMammogram intervention? What did you think of the content of the mMammogram program? Was it helpful to improve your knowledge in breast cancer screening? and What are your suggestions for wide dissemination of the mMammogram intervention in our community? All focus groups were conducted in Korean. Each focus group lasted approximately 1–1.5 h and was digitally recorded. Written consent was obtained from all 14 participants before starting the focus groups. Each participant received $20 for her time commitment.

## 3. Data Analysis

A thematic analysis [29] was used to analyze the data and to identify the essential themes discussed. The thematic analysis involves six steps: (1) being familiar with written transcription of verbal data; (2) generating initial codes; (3) searching and deciding on a set of codes, subcategories, and categories; (4) identifying themes and reviewing the themes; (5) defining and naming themes; and (6) producing the report. To follow the steps, first, all audio interview files were transcribed in Korean by three bilingual Korean research assistants and then reviewed by the research team. Second, two of three bilingual Korean research assistants coded all transcripts separately. Each person identified and highlighted every codable unit of text in the transcripts and compared their analyses (codes, subcategories, and categories). Based on the codes, the research team established and reviewed themes. Themes were cross-checked to ensure they were representative of the transcripts. Clear definitions and names for each theme were generated through the process. Finally, the most representative quotes were selected to present in this paper and translated into English. Final translations were examined by all authors who are bilingual and bicultural.

## 4. Findings

### 4.1. Study Participants

Table 1 indicates study participants’ sociodemographic characteristics. The mean age of all participants was 50.57 years old (Standard Deviation (SD) = 6.64). On average, they lived in the U.S. for 14 years (SD = 9.71). The majority of the participants (92.9%) were married. With respect to educational background, half of the participants (50%) had completed university and around 42.9% finished high school. Approximately 64.3% were currently employed. More than two-thirds of the participants (71.4%) had health insurance and only 35.7% had a primary healthcare provider. Only 14.3% had regular check-ups. Around 28.6% of the participants reported that their most recent mammogram was before 2010 and 35.7% had never had a mammogram in their life.

### 4.2. Themes from Focus Groups

Overall, three themes were identified from the focus groups: (1) better understanding of breast cancer and screening; (2) health navigators as a trigger to promote mammography; and (3) suggestions for mMammogram. We indicated each participant’s number when describing quotes to protect the confidentiality of each participant. 

#### 4.2.1. Theme 1. Better Understanding about Breast Cancer and Mammography

Study participants reported that mMammogram helped to (1) increase their awareness, positive attitude, and knowledge on breast cancer and breast cancer screening methods; (2) increase their understanding of the importance of regular mammography; and (3) reduce their anxiety about mammography, resulting in promoting their interests in screening participation.

(1) Increased awareness and knowledge on breast cancer and screening methods

Overall, almost all participants (*n* = 12) stated that mMammogram was helpful to learn basic knowledge of breast cancer, as well as the three breast cancer screening methods: breast self-exam, clinical breast exam, and mammogram. Several participants reported that they were unaware of how prevalent breast cancer is in the Korean American community, which naturally led to their limited awareness about breast cancer screening. Interestingly, after participating in the program, they started to pay attention to information related to breast cancer when they watched TV, listened to the radio, or had conversations with their friends. The participants reported that mMammogram makes them take breast cancer seriously and encourages them to consider getting a mammogram. For example, participant #1 decided to get a mammogram after completing the program:

“You know… I was surprised by the fact that breast cancer is common in our community. I believed that it is a rare disease in our community. When people talk about breast cancer, I did not carefully listen to [them]. However, the statistical graph related to breast cancer incidence and mortality in Korean American women caught my attention. It makes me to consider it more serious, leading me to think about mammogram. Early detection is better, right?”

In addition, seven participants explained how their misperceptions about mammograms were corrected through mMammogram. The common misperceptions reported by the study participants included (1) people who are older do not need screening and (2) mammogram causes radiation exposure, which also may cause cancer. Four participants knew an older woman with breast cancer and they thought it was a very rare case, indicating that older women do not need to get screened. For example, one participant admitted that she made a joke about her 89-year old mother-in-law who got a mammogram. She could not understand why her old mother-in-law got a mammogram at that time, but she was able to understand after completing mMammogram. Now she knows that old age is a risk factor for breast cancer and that women should get a mammogram regardless of their age. Three other participants expressed their concern about radiation exposure; their concern even led them to not get a mammogram. For instance, participant #3 shared her story:

“I used to get a mammogram every year or every other year… and my last mammogram was in 2011. I have several reasons to stop getting it. One of the reasons is possible harm from the radiation exposure during mammogram. I worried about cumulative radiation exposure for several years because radiation exposure could be a risk factor for breast cancer, you know. This program highlights the early detection of mammogram. Plus, a female doctor in the video said that mammogram only involves a tiny amount of radiation and the benefits of mammogram outweigh possible harm from the radiation exposure. I am fully aware of it now, which led me get a mammogram again. If I did not participate in this study, I would not get a mammogram for many years.”

(2) Reminder of the importance of regular mammography

Some participants (*n* = 5) did not understand why women should get a mammogram on a regular basis. They had received a mammogram before, but they did not adhere to routine screening for several reasons, such as having no symptoms on their breasts, having a busy life, and having health insurance issues. For example, participant #6 did not follow her physician’s recommendation. She was young and healthy, so she could not find reasons to get a mammogram again, even with her physician’s continuous suggestion:

“Whenever I saw a doctor, he recommended me to get a mammogram because my last mammogram was 7 years ago. However, I ignored his suggestion. You know, I am healthy enough. No symptoms on my breasts. Why should I get a mammogram? Plus, I have conducted breast self-exam and had clinical breast exam. I thought these two methods are enough to check my breast. However, my thoughts have changed after I completed this program. I realized that I needed to get a mammogram on a regular basis, regardless of my health status. I learned that the two methods are not sufficient to find cancer. I did not know that breast cancer is very common in Korean American women, which makes me feel anxious.”

The participants reported that mMammogram was helpful in reminding them about their breast health and increasing their awareness of getting regular mammography. For instance, participant #2 said that she made a schedule to get a mammogram after completing the program. She used to get mammograms in Korea because of her breast symptoms, but she stopped receiving them after she came to the U.S. because her life was too busy and she did not feel the symptoms anymore. mMammogram reminded her that she should continue to care for her breasts. 

(3) Reduced anxiety about mammography

Half of the participants (*n* = 7) reported that mMammogram—particularly, two videos about mammogram procedures—helped reduce their anxiety about breast cancer screening. The participants were highly satisfied with the videos that demonstrated the procedure of a mammogram. One video showed the mammogram procedure briefly. The other video showed a technician at a breast imaging center explaining the mammogram procedure in step-by-step detail, from the check-in process at the front desk to taking images of the breasts. The participants mentioned that they felt a bit more confident in getting a mammogram after watching the videos. They reported getting a sense of the whole procedure of mammography, which resulted in relieving their anxiety about going to a hospital to get a mammogram. For example, participant #14 said:

“I felt safe to get a mammogram after watching two videos about mammogram procedure. I learned that I don’t need to take off all my clothes while imaging my breasts… just exposure one side of breast and then if it is done… move to the other side of breast. You know the video. A nurse showed the rooms where we can change our clothes and where the mammogram machine is. This all information was super helpful to relieve my anxiety about mammogram. Now I know what to expect when I go to hospital to get a mammogram”

More than half of the study participants (*n* = 8) showed passion for learning more about breast cancer, beyond what was covered in the program, such as breast density and its relationship with breast cancer incidence and various cases of breast cancer by stage and related treatment options. In addition, they were highly motivated to educate other women about the importance of breast cancer screening, including colleagues at work, friends, and family members. They wanted to share what they learned from the program with other women in the community who did not or were not able to participate in this study. Two participants said that, after completing mMammogram, they partly showed the program to their daughters to educate them on how to conduct a breast self-exam:

“I showed a part of the mMammogram application (app) program to my daughter, in particular the breast self-exam video. She is a college student and I think she needs to start self-examination.”

(Participant #3)

#### 4.2.2. Theme 2. Health Navigators as a Trigger to Promote Mammography

As a part of mMammogram, health navigation services were offered to the study participants. The services included obtaining necessary resources (e.g., free or low-cost mammograms) for participants who did not have health insurance, scheduling mammogram appointments for participants, helping participants select a primary care physician, helping fill out forms at hospitals or clinics, transporting participants to receive a mammogram, and/or following up after receiving a mammogram for those who have abnormal results. The study participants had an option to contact bilingual health navigators to support them in getting a mammogram. The most common requests were checking whether their health insurance covered mammography and if they were eligible to get a mammogram for free or at low cost. 

(1) Importance of Health Navigation Services 

Some of the focus group participants (*n* = 9) reported difficulties accessing healthcare due to their limited English proficiency. They highlighted how bilingual health navigators were helpful in supporting their accessibility, resulting in increased screening uptake. Some participants (*n* = 5) mentioned the complexity of health insurance. For example, participant #4 reported that her company chooses different health plans every year, which presents more challenges to getting a mammogram:

“All the problem is rooted in health insurance. You know what? My health insurance plan is changed every year, which led me discourage to access health care. It used to change every two or three years. (…). The coverage is getting worse. I need to find a new family doctor again... and check my coverage which I do feel uncomfortable because of my English. I am getting tired of it. I sometimes think to visit Korea to get comprehensive health exam. That is going to be much easier and cheaper.”

(2) Health Navigation Services and Regular Mammogram Receipt

The study participants reported how bilingual health navigators helped them overcome barriers to screening (e.g., health insurance and communication with healthcare providers). They also indicated intentions to continue getting mammograms if they are able to access a health navigation service. For instance, participant #1 shared her story:

“After participating in this program, I decided to get a mammogram. It was my first mammogram in my lifetime. I was nervous and worried about receiving mammogram because I am not familiar with U.S. health care system and my English is not enough to communicate with doctor or nurse. I believed that I was able to get a mammogram because of health navigator’s support. She called my health insurance company to check my insurance coverage. And then, she scheduled a mammogram appointment for me and went to the clinic with me. Such a good service. I really appreciated all the things she did. You know, I would like to continue to get mammography every year if someone reminds me and assists me to get it.”

#### 4.2.3. Theme 3. Suggestions for Improvement and Dissemination of the mMammogram 

Although study participants were highly satisfied with mMammogram, they suggested how the intervention could be improved for wider dissemination and implementation in the Korean American community. Their suggestions include larger subtitles on videos, addressing technical issues (e.g., Wi-Fi connection), extended schedule of message delivery, and adjusting the program period duration. For example, a total of eight videos were provided via mMammogram that included Korean or English subtitles. Some participants (*n* = 8) said that the subtitles on videos were too small to read via their phone, even though they were wearing glasses. They had to watch the videos several times to read the subtitles and understand the contents. Second, sometimes participants faced challenges to using their phone, such as issues with Wi-Fi connection. In addition, participants expressed a desire to receive messages later at night or at other times based on their schedules. In the current mMammogram system, study participants could select their preferred time to receive the messages between 8 a.m. and 8 p.m. They suggested they could control the starting time of the program, given that their schedules could change from day to day. Otherwise, they were unable to attend the program on time and missed it. Lastly, participants had different opinions regarding the program period. Some participants suggested a shorter period of 3–5 days, while others preferred a 7-day program. 

## 5. Discussion

This study explored (1) how mMammogram helped Korean American immigrant women to increase their knowledge and positive attitude toward mammography; (2) how the program motivated them to complete breast cancer screening; and (3) how the program could be improved and disseminated in the Korean American community. Along with these findings, this study demonstrated that a mobile phone-based multimedia health intervention with health navigation services can be a feasible, effective, and acceptable tool to promote mammography uptake in underserved immigrant women. 

We identified three themes: (1) better understanding of breast cancer and screening through mMammogram intervention; (2) importance of health navigator services to promote mammography, and (3) suggestions for mMammogram improvement and dissemination strategies. As noted in Theme 1, multimedia messages (e.g., images, pictures, and videos) through a mobile phone-based application have the advantage of being entertaining and individual-targeted to capture study participants’ attention. For example, video content held great persuasive power in our intervention. Video messages were the most popular and well-accepted among the study participants, and they remembered the contents conveyed via videos much clearer and longer than other text messages. In particular, study participants liked two video clips that featured mammogram procedures and the importance of having a regular mammogram. Those who were unfamiliar with U.S. hospitals and who never had a mammogram previously got a sense of what would happen when they go to a clinic for mammography. This improved their confidence about mammography and promoted their interest in screening participation [30,31].

Another merit of a mobile phone-based health intervention is its easy dissemination, accessibility, and portability by having important health information at the user’s fingertips. Plus, the study participants can keep the program if they want and open it anytime to remind themselves or inform other people. A few study participants who had a passionate interest in our program taught their daughters how to conduct a breast self-exam by showing them the videos from the program. This was an unintended impact of our intervention, which implies ”ripple effects” in that they led to new effects beyond the intended primary target of the intervention [32]. The participants’ daughters may now have increased awareness of breast cancer screening at an early age and may start their mammography in the right time frame. 

As discussed in Theme 2, it is well-known that immigrants face various challenges in navigating the U.S. healthcare system due to lack of health insurance literacy and underutilization of healthcare services due to limited English proficiency. In particular, undocumented immigrants’ barriers range from financial issues to fear of deportation [33]. Health navigation models of care have been employed as a strategy to support individuals with limited resources and multiple barriers, and health navigators have made significant differences to health equity in culturally and linguistically diverse communities [34,35,36]. Our study also indicated how health navigation services played critical roles in boosting the participants’ accessibility to receiving breast cancer screenings. Study participants could contact trained health navigators while attending mMammogram. They felt assured that they could ask for help in their own language as their needs increased. With navigators’ assistance, some participants had a mammogram for the first time in their lives. Plus, they showed their intention to continue getting a mammogram if a health navigation service offers. Given that the U.S. healthcare system is complicated, and the healthcare environment continues to change, health navigator services and educational programs could be employed as tools to reduce breast cancer disparities.

As seen in Theme 3, on the other hand, several challenges were reported while attending mMammogram intervention. One of the challenges of a mobile phone-base health intervention is the various sizes and types of smartphones. Some participants had the latest phones with big screens (5.5 inches or larger), while some had older versions of mobile phones. Those who had mini smartphones had difficulty reading the subtitles on the videos. Given that the size of participants’ phones cannot be controlled, we may consider enlarging the subtitles or dubbing the video in participants’ native language. We need to further consider developing an application that could be downloaded onto the iPad, where participants may read subtitles through a large window. Additionally, some participants faced challenges in using their phones due to weak Wi-Fi connections or a complicated process of downloading an app. They still felt challenged even after our research team taught them how to access to our app program and respond to it before the intervention. With only a short time for training, it might be hard to overcome all challenges faced by study participants. We may need to provide ongoing training via health navigation services or a handout on how to deal with common technical challenges. Another solution is to develop a web-based app which does not need to be downloaded and can be easily seen by smartphones and any other mobile devices. 

## 6. Limitations

This study provides a valuable contribution to knowledge on how mHealth can be used to change health behavior, particularly for promoting breast cancer screening; however, some limitations may affect its applicability to other contexts. First, while a small sample size is not a limitation of qualitative research, the data were collected from one-time focus group meetings. However, the examination of the study participants’ responses to open-ended questions regarding evaluation of the intervention after the completion of mMammogram facilitated further insights into the evaluation of and feedback on a mobile-phone text-messaging program. Lastly, the frequent interactions between study participants and research team members may impact participants’ knowledge and motivation to receive a breast cancer screening, as well as their positive attitudes toward the intervention. Whenever study participants had questions related to the app (e.g., technical issue) or contents, they were allowed to contact us, which may positively impact intervention effectiveness and study results.

## 7. Implications for Health Practice and Policy

In conclusion, mobile apps can cover a broad range of breast cancer health topics, and the health navigator can further help women overcome barriers to screening. Specifically, for newly arrived immigrant populations, a health navigation service is critical in overcoming language, transportation, and health accessibility barriers and triggering a positive change in their health screening behavior. This study demonstrates the effectiveness of a culturally tailored mobile app intervention in increasing knowledge, attitude, and receipt of breast cancer screening in an immigrant group. Mobile app intervention should be combined with health navigation services, particularly when working with immigrant or refugee groups, to provide another layer of assistance which boosts confidence and comfort level in changing health behaviors. In addition, it was revealed that providing educational health information through visual images and videos is more effective than text messages. Overall, culturally and linguistically tailored multimedia messaging intervention combined with health navigation services is feasible and acceptable as an intervention tool to produce health behavior changes. The intervention can be easily translated into other types of cancer screening and cancer prevention behaviors, which will significantly reduce the current cancer burden vulnerability that immigrant and refugee communities currently face.

## 8. Conclusions

By applying the mobile phone-based multimedia messaging program, the most prominent findings include better understanding of breast cancer and screening through mMammogram intervention and the importance of health navigator to promote mammography, as well as suggestions for mMammogram improvements and dissemination strategies. Identifying these three themes provided important insights for mMammogram intervention to increase the breast cancer screening. The vital role of these three implies the mobile phone-based multimedia messaging program may be useful for reducing multiple challenges and barriers in navigating the healthcare system and increasing the level of health literacy implemented in the immigrant population. 

In light of the present findings, to produce health behavior changes and mitigate challenges in the healthcare system, mobile apps must cover a wide range of health topics in different languages with health navigator services. Mobile apps should also be tailored to different cultures among the immigrant populations to trigger positive changes in increasing health literacy and receipt of cancer screening. Furthermore, the tailored multimedia messaging intervention also need to combine with visual images and videos. In sum, culturally and linguistically tailored multimedia messaging intervention combined with health navigation services is useful and effective to reduce the current cancer burden vulnerability faced by immigrant populations.

## Figures and Tables

**Table 1:** The sociodemographics of study participants.

	Age	Years in the U.S.	Marital Status	Highest Education Level	Employment Status	Health Insurance	Primary Health Care Provider	Regular Check-Up	Recent Mammogram
1	44	5	Married	High school	Employed	Yes	No	No	Never screened
2	60	3	Separated or divorced	High school	Employed	Yes	No	Yes	2012
3	57	5	Married	High school	Employed	Yes	Yes	No	2011
4	51	13	Married	University	Employed	Yes	Yes	Yes	2003
5	50	14	Married	High school	Employed	No	Yes	No	Never screened
6	45	6	Married	High school	Employed	No	No	No	2008
7	53	13	Married	High school	Unemployed	No	Yes	No	2012
8	52	28	Married	University	Employed	Yes	No	No	2009
9	48	20	Married	University	Unemployed	Yes	No	No	2009
10	46	4	Married	University	Employed	Yes	No	No	2010
11	58	14	Married	University	Unemployed	No	No	No	Never screened
12	40	12	Married	University	Unemployed	Yes	No	No	Never screened
13	61	35	Married	University	Unemployed	Yes	Yes	No	2011
14	43	24	Married	Graduate school	Employed	Yes	No	No	Never screened
